# Impact of Serum Proteins on the Uptake and RNAi Activity of GalNAc-Conjugated siRNAs

**DOI:** 10.1089/nat.2020.0919

**Published:** 2021-08-04

**Authors:** Saket Agarwal, Ruth Allard, Justin Darcy, Samantha Chigas, Yongli Gu, Tuyen Nguyen, Sarah Bond, Saeho Chong, Jing-Tao Wu, Maja M. Janas

**Affiliations:** Early Development, Alnylam Pharmaceuticals, Inc., Cambridge, Massachusetts, USA.

**Keywords:** siRNA, RNAi, GalNAc conjugate, protein binding, *ASGPR*

## Abstract

Serum protein interactions are evaluated during the drug development process since they determine the free drug concentration in blood and thereby can influence the drug's pharmacokinetic and pharmacodynamic properties. While the impact of serum proteins on the disposition of small molecules is well understood, it is not yet well characterized for a new modality, RNA interference therapeutics. When administered systemically, small interfering RNAs (siRNAs) conjugated to the *N*-acetylgalactosamine (GalNAc) ligand bind to proteins present in circulation. However, it is not known if these protein interactions may impact the GalNAc-conjugated siRNA uptake into hepatocytes mediated through the asialoglycoprotein receptor (*ASGPR*) and thereby influence the activity of GalNAc-conjugated siRNAs. In this study, we assess the impact of serum proteins on the uptake and activity of GalNAc-conjugated siRNAs in primary human hepatocytes. We found that a significant portion of the GalNAc-conjugated siRNAs is bound to serum proteins. However, *ASGPR*-mediated uptake and activity of GalNAc-conjugated siRNAs were minimally impacted by the presence of serum relative to their uptake and activity in the absence of serum. Therefore, in contrast to small molecules, serum proteins are expected to have minimal impact on pharmacokinetic and pharmacodynamic properties of GalNAc-conjugated siRNAs.

## Introduction

Small interfering RNAs (siRNAs) are a new class of emerging RNA interference (RNAi) therapeutics with the potential to treat a multitude of debilitating diseases. Recently, three siRNA therapeutics have been approved by the Food and Drug Administration (FDA)—ONPATTRO^®^ (patisiran) in 2018, GIVLAARI^®^ (givosiran) in 2019, and OXLUMO™ (lumasiran) in 2020. ONPATTRO is the first-in-class, lipid nanoparticle-based RNAi drug approved by the FDA for the treatment of polyneuropathy caused by hereditary transthyretin amyloidosis [1]. GIVLAARI is the first *N*-acetylgalactosamine (GalNAc)-conjugated siRNA approved by the FDA for the treatment of acute hepatic porphyria in adults [2]. Another GalNAc-conjugated siRNA, OXLUMO, was recently approved by the FDA for the treatment of primary hyperoxaluria type 1.

The GalNAc ligand is used to specifically target siRNA delivery to the liver since it binds with high affinity to the asialoglycoprotein receptor (*ASGPR*), which is predominantly and highly expressed on the surface of hepatocytes [3,4]. Following endocytosis through clathrin-coated vesicles, the siRNA is released from acidic endosomal compartments into the cytosol and loads into the RNA-induced silencing complex (RISC) [5,6]. Watson and Crick base pairing between the loaded siRNA and its target messenger RNA (mRNA) leads to catalytic cleavage of the mRNA, mediated by RISC. In recent years, significant improvements in siRNA chemistry have led to the development of enhanced stabilization chemistry (ESC) and advanced ESC designs [7]. The ESC design of GalNAc-conjugated siRNAs described here is representative of GalNAc-conjugated siRNAs that are currently in various stages of clinical development across multiple therapeutic areas.

Serum protein interactions are routinely evaluated during the small-molecule drug development process since they determine the free drug concentration in circulation, which influences the drug's pharmacokinetic and pharmacodynamic properties. The pharmacological activity of conventional small-molecule drugs is generally mediated by unbound drug, not the total drug, and thus the extent of protein binding is routinely measured at equilibrium [8,9]. GalNAc-conjugated siRNAs are bound to proteins in circulation, and recent publications have described methods to quantify the extent of protein binding using ultrafiltration [10] and the electrophoretic mobility shift assay (EMSA) [11]. At clinically relevant concentrations (Cmax ≤1 μg/mL [12]), percent of GalNAc-conjugated siRNA bound to plasma proteins reaches up to 90%, and at concentrations ≥1 μg/mL, percent of GalNAc-conjugated siRNA that is bound decreases as serum protein binding saturates [11]. These observations are consistent across nonclinical species *in vitro*, where serum protein binding to GalNAc-conjugated siRNAs is dependent on siRNA and serum concentrations [11]. However, the impact of serum proteins on GalNAc-conjugated siRNA uptake and activity in hepatocytes has not yet been studied.

To understand the effect of serum proteins on *ASGPR*-mediated uptake and activity of GalNAc-conjugated siRNAs, we utilized a primary human hepatocyte free uptake assay in the presence or absence of human serum *in vitro*. Intracellular GalNAc-conjugated siRNA concentrations were measured to assess the impact on uptake, and reduction in target mRNA was measured to assess the impact on RNAi activity.

## Materials and Methods

### Test materials and oligonucleotide synthesis

All GalNAc-conjugated siRNAs were synthesized by Alnylam Pharmaceuticals (Cambridge, MA, USA). Three GalNAc ligands were covalently linked to the 3′ end of the sense strand of the siRNA by a phosphodiester linkage between the pyrrolidine scaffold, as described previously [13,14]. All the information pertinent to the tested GalNAc-conjugated siRNAs can be found in Table 1.

**Table 1.** Designs, Sequences, and Target Messenger RNAs of the Small Interfering RNAs Used in This Study

Compound	Strand	Sequence (5′–3′)	Target	Target accession number
AD-60519	S	c•a•gaaaGaGuGuCuCaucuuaL	*ALAS1*	NM_000688
	AS	u•A•AGaUgAgAcAcUcUuUcUg•g•u		
siRNA2	S	Proprietary^a^	*AAT*	NM_001127701
	AS	Proprietary^a^		
AD-65644	S	g•a•auguGaaAGucaucgacaaL	*HAO1*	NM_017545
	AS	u•U•gucGaUGacuuUcAcauuc•u•g		
AD-64543	S	c•a•CuGuGaCUGuGgCcUcCaAL	*TMPRSS6*	NM_153609
	AS	u•U•gGaGgCcAcagUcAcAgUg•c•u		
AD-68435	S	u•g•ugcaAuGAAaggcaaauauL	*F9*	NM_000133
	AS	a•U•auuUgCCuuucAuUgcaca•c•u		

**L** 
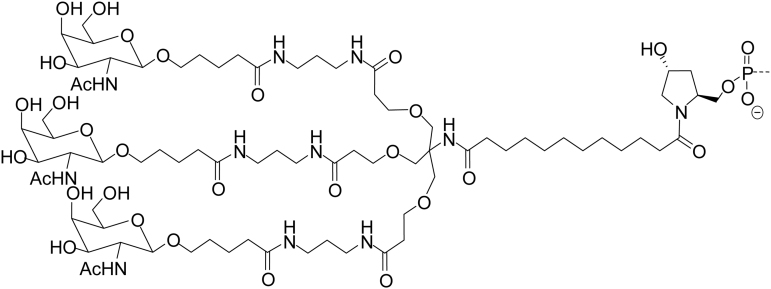

S and AS represent sense and antisense strands; uppercase and lowercase letters indicate 2′-deoxy-2′-fluoro (2′-F) and 2′-*O*-methyl (2′-*O*Me) ribosugar modifications, respectively.

^a^ Design similar to GalNAc-conjugated siRNAs described in this table; • indicates phosphorothioate linkage; **L** indicates the trivalent GalNAc ligand (structure above).

*AAT*, alpha-1 antitrypsin; *ALAS1*, aminolevulinic acid synthase; *F9*, coagulation factor IX; GalNAc, *N*-acetylgalactosamine; *HAO1*, hydroxy acid oxidase 1; siRNA, small interfering RNA; *TMPRSS6*, transmembrane serine protease 6.

### Serum incubation for protein binding measurement

GalNAc-conjugated siRNAs were added to INVITROGRO™ HI Medium (Cat. No. Z99009; BioIVT) supplemented with 100%, 80%, 60%, 40%, 20%, or 0% fetal bovine serum (FBS; Cat. No. 10082147; Gibco) or 60% human serum (Cat. No. HUMANSRMMNN; BioIVT;) and incubated for 60 min at 37°C. The percent of siRNA bound to serum proteins was assessed using the EMSA described previously [11]. In short, the samples were prepared by adding 6 × EMSA gel-loading solution and loaded onto a 10% Tris-borate-EDTA (TBE) gel. The gel was run at 100 V, on ice, stained with SYBR(R) gold nucleic acid gel stain, washed with TBE, imaged, and analyzed using the Gel Doc XR+ System with Image Lab software, version 5.2. The intensity of the bound siRNA was compared with the control (no serum) band run on the same gel. Percent binding was calculated from the measured intensity ratios.

### Primary hepatocyte cell culture and treatment

Primary human hepatocytes (Cat. No. F00995-P; BioIVT) were seeded in 24-well or 96-well collagen I precoated plates (Cat. No. A1142803; Gibco) at ∼0.2 × 10^6^ cells/well in 500 μL or 5 × 10^4^ cells/well in 95 μL of INVITROGRO HI medium (Cat. No. Z99009; BioIVT), respectively, supplemented with 60%, 40%, 10%, or 0% human serum (Cat. No. HUMANSRMMNN; BioIVT). Twenty-five microliters of GalNAc-conjugated siRNA duplex (at 20 × final concentration) per well was added to cells to yield 1 × final concentration in 24-well plate experiments. Five microliters of GalNAc-conjugated siRNA duplex (at 20 × final concentration) per well was added to cells to yield 1 × final concentration in 96-well plate experiments. Cells were incubated for 4 h at 37°C under these conditions. After the 4-h incubation, the media were removed and replaced with serum- and siRNA-free medium, 500 μL (24-well plate) or 100 μL (96-well plate), and incubated for 48 h at 37°C. The cells from 24-well plates were processed to quantify siRNA concentration, and the cells from 96-well plates were processed to isolate total RNA.

### siRNA quantification

Primary human hepatocytes cultured in 24-well plates were washed with phosphate-buffered saline (PBS) and lysed with 250 μL of ice-cold lysis buffer (0.25% Triton X in PBS). A 9-point standard curve was generated for each siRNA using the lysis buffer as a diluent. Diluted samples (1:100 in lysis buffer) and standards were heated at 95°C for 10 min. Immediately following heat denaturation, 5 μL of each sample was added directly to 10 μL of a stem–loop reverse transcription (RT) Master Mix (Cat. No. 4366597; Applied Biosystems™ TaqMan™ MicroRNA Reverse Transcription Kit). The reactions were performed using the following parameters: RT (16°C for 30 min, followed by 42°C for 30 min) and the stop reaction (85°C for 5 min). The resulting complimentary DNA (cDNA) was diluted in lysis buffer to a final volume of 37.5 μL. Quantitative polymerase chain reaction (qPCR) was carried out using an ABI ViiA 7 and QuantStudio software, using an Applied Biosystems TaqMan Fast Advanced Master Mix (Cat. No. 4444557) in 20-μL reactions. The cycling parameters for the PCR were as follows: uracil-DNA glycosylase incubation (50°C for 2 min), polymerase activation (95°C for 2 min), and 40 cycles of PCR with a denaturing step (95°C for 1 s) and an annealing/extension step (60°C for 20 s). The average cycle time (Ct) values of the 9-point standard curve were then used to form a linear regression to calculate the concentration of the siRNA present in each sample. The stem–loop primer, forward primer, reverse primer, and probe sequences are listed in Table 2.

**Table 2.** Reverse Transcription–Quantitative Polymerase Chain Reaction Primer and Probe Designs for Small Interfering RNA Quantification

siRNA	Primer	Sequence (5′–3′)
AD-60519	Stem–loop	GTCGTATCCAGTGCAGGGTCCGAGGTATTCGCACTGGATACGACACCAGAAAGA
	Forward qPCR	GCCGCGCTAAGATGAGACA
	TaqMan probe	CTGGATACGACACCAGAAA
siRNA2	Stem–loop	Proprietary
	Forward qPCR	Proprietary
	TaqMan probe	Proprietary
AD-65644	Stem–loop	GTCGTATCCAGTGCAGGGTCCGAGGTATTCGCACTGGATACGACCAGAATGTGA
	Forward qPCR	GCCGTTGTCGATGACTTT
	TaqMan probe	TGGATACGACCAGAATG
AD-64543	Stem–loop	GTCGTATCCAGTGCAGGGTCCGAGGTATTCGCACTGGATACGACAGCACTGTGA
	Forward qPCR	GCTTGGAGGCCACAGTC
	TaqMan probe	TGGATACGACAGCACT
AD-68435	Stem–loop	GTCGTATCCAGTGCAGGGTCCGAGGTATTCGCACTGGATACGACAGTGTGCAATG
	Forward qPCR	GCCGCGCATATTTGCCTTTC
	TaqMan probe	CTGGATACGACAGTGTGC
Universal	Reverse qPCR	GTGCAGGGTCCGAGGT

qPCR, quantitative polymerase chain reaction.

### RNA isolation

RNA was isolated from primary human hepatocytes cultured in 96-well plates using the RNAqueous™-96 Total RNA Isolation Kit (Cat. No. AM1920; Ambion) following the manufacturer's protocol. Briefly, 200 μL of lysis/binding solution was added to the cells and pipetted up and down several times to obtain a homogeneous lysate. One hundred microliters of 100% ethanol was added, mixed, and passed through the wells of the filter plate. The filters were washed with 300 μL of wash solution and passed through the filter plate. DNase I working solution was prepared and 20 μL was added to the center of the well in the filter plate. The plate was incubated at room temperature for 20 min. After incubation, 240 μL/well of rebinding mix was added and passed through the filter plate. The filter plate was washed twice, once with 300 μL and once with 200 μL of wash solution. After the wash solution had passed, the plate was centrifuged at 1,500*g* for 2 min. RNA was eluted in 60 μL of nuclease-free water by centrifuging at 1,500*g* for 2 min.

### Reverse transcription–quantitative polymerase chain reaction

cDNA was synthesized using the Applied Biosystems High-Capacity cDNA Reverse Transcription Kit (Cat. No. 4368813; Applied Biosystems). Briefly, 10 μL of a master mix containing 1 μL of 10 × buffer, 0.4 μL of 25 × deoxynucleoside triphosphates, 1 μL of 10 × random primers, 0.5 μL of MultiScribe™ reverse transcriptase, 0.5 μL of RNase inhibitor, and 6.6 μL of nuclease-free water per reaction was added to 10 μL of the isolated RNA. Plates were incubated in a thermal cycler programmed for four steps: step 1—25°C for 10 min, step 2—37°C for 120 min, step 3—85°C for 5 min, and step 4—hold at 4°C.

cDNA was diluted with 130 μL of nuclease-free water and 4.5 μL of the resultant mix was added to a master mix containing 0.5 μL of actin, beta (*ACTB*) TaqMan probe (Cat. No. Hs99999903_m1; Thermo Fisher Scientific), and 5 μL of LightCycler^®^ 480 probe master mix (Cat. No. 04887301001; Roche) per well in a 384-well plate (Cat. No. 04729749001; Roche) for each treatment. Similarly, in a separate set, 4.5 μL of cDNA was added to 0.5 μL of respective target transcript probe (Table 3) for each GalNAc-conjugated siRNA treatment, along with 5 μL of LightCycler 480 probe master mix. qPCR was carried out with the LightCycler 480 Real-Time PCR System (Roche) using the Ct(relative quantification) assay. Each treatment was tested in three independent replicates. To calculate relative fold change, real-time qPCR data were analyzed using the Ct method. On-target mRNA values were normalized to *ACTB* values for each treatment, and data were presented as percent change relative to PBS-treated (mock) cells.

**Table 3.** Reverse Transcription–Quantitative Polymerase Chain Reaction Primer and Probe Designs for Messenger RNA Knockdown Assessment

siRNA	Target	Catalog no. (Thermo Fisher Scientific)
AD-60519	*ALAS1*	Hs00963537_m1
siRNA2	*AAT*	Hs00165475_m1
AD-65644	*HAO1*	Hs00213909_m1
AD-64543	*TMPRSS6*	Hs00542191_m1
AD-68435	*F9*	Hs01592597_m1

## Results

### Percent of GalNAc-conjugated siRNA bound to serum proteins

We first sought to understand how the fraction of GalNAc-conjugated siRNA bound to proteins changes as a function of serum concentration. A GalNAc-conjugated siRNA (AD-60519) at 4 μg/mL (250 nM) was incubated with increasing amounts of FBS (0%, 20%, 40%, 60%, 80%, or 100%, v/v) for 60 min at 37°C, and the percent of GalNAc-conjugated siRNA that is bound to serum proteins was measured using the EMSA [11]. The percent binding to serum proteins increased with increasing FBS concentrations (Table 4), reaching ∼82% bound (or 18% unbound) in neat serum conditions. Additionally, we assessed protein binding interactions of two other representative GalNAc-conjugated siRNAs (AD-65644 and AD-64543) at ∼3.2 μg/mL (200 nM) in the presence of 60% human serum. This concentration was chosen since GalNAc-conjugated siRNAs evaluated here are expected to show reasonable *in vitro* activity at this concentration, although the potency of each siRNA may vary. Consistent with the FBS results, ∼65%–70% of GalNAc-conjugated siRNAs were bound to proteins in human serum under these conditions (Table 4). Based on these data, we chose to assess the uptake and RNAi activity of GalNAc-conjugated siRNAs incubated with up to 60% human serum.

**Table 4.** Percent of *N*-acetylgalactosamine-Conjugated Small Interfering RNA Bound to Proteins in Varying Concentrations of Fetal Bovine or Human Serum

GalNAc-conjugated siRNA	Serum	20%	40%	60%	80%	100%
AD-60519	Bovine	11.7 ± 7.0	58.4 ± 6.9	71.4 ± 3.4	75.4 ± 1.8	81.8 ± 2.8
AD-65644	Human	NT	NT	65.5 ± 1.2	NT	NT
AD-64543	Human	NT	NT	71.8 ± 0.6	NT	NT

NT, not tested.

### Impact of serum proteins on *in vitro* uptake of GalNAc-conjugated siRNAs

**FIG. 1.**
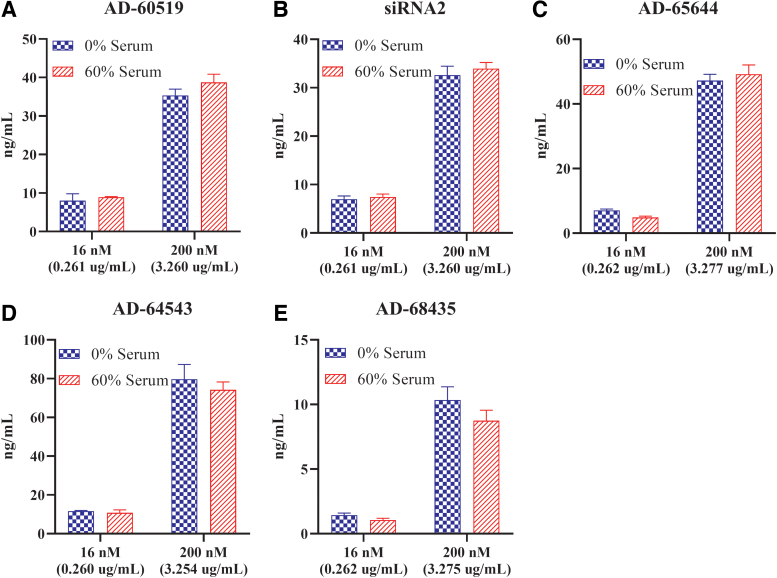
Minimal impact of human serum proteins on GalNAc-conjugated siRNA uptake in human hepatocytes. Primary human hepatocytes were treated with 16 nM (∼0.26 μg/mL) or 200 nM (∼3.2 μg/mL) AD-60519 **(A)**, siRNA2 **(B)**, AD-65644 **(C)**, AD-64543 **(D)**, or AD-68435 **(E)** by free uptake in the absence of human serum or presence of 60% human serum. After 48 h of incubation, the siRNA level in the lysate was assessed by stem–loop RT-qPCR and absolute values obtained were plotted. Each bar represents the average of three replicates. Error bars represent standard deviation. GalNAc, *N*-acetylgalactosamine; RT-qPCR, reverse transcription–quantitative polymerase chain reaction; siRNA, small interfering RNA. Color images are available online.

Since a significant percent of GalNAc-conjugated siRNAs were bound to serum proteins, we next studied the impact of serum proteins on GalNAc-conjugated siRNA uptake into human hepatocytes through the *ASGPR*. At ∼0.26 μg/mL (16 nM) and ∼3.2 μg/mL (200 nM) concentrations, GalNAc-conjugated siRNAs were tested in the absence human serum or presence of 60% human serum in the media. After 4 h of incubation to allow free uptake of GalNAc-conjugated siRNAs through the *ASGPR*, the media were replaced with serum- and siRNA-free media for 48 h, followed by siRNA quantification. As expected, in the absence of human serum, there was a concentration-dependent increase in siRNA concentration with all five compounds tested (Fig. 1A–E). When incubated with 60% human serum, AD-65644 and AD-64543 at ∼3.2 μg/mL (200 nM) were ∼65% and 70% bound (or 35% and 30% unbound) to serum proteins, respectively (Table 4). When primary human hepatocytes were treated with the same concentration of AD-65644, ∼47 ± 2 ng/mL siRNA was measured without human serum and ∼49 ± 3 ng/mL siRNA was measured with 60% human serum (Fig. 1C). Similarly, ∼3.2 μg/mL (200 nM) AD-64543 treatment in primary human hepatocytes in the absence of human serum incubation led to an ∼80 ± 0.4 ng/mL siRNA measurement, and treatment with 60% human serum led to ∼74 ± 1.5 ng/mL measurement (Fig. 1D). Thus, in both cases, the *ASGPR*-mediated uptake of GalNAc-conjugated siRNAs into hepatocytes through the *ASGPR* was minimally impacted by serum proteins.

### Impact of serum proteins on *in vitro* RNAi activity of GalNAc-conjugated siRNAs

**FIG. 2.**
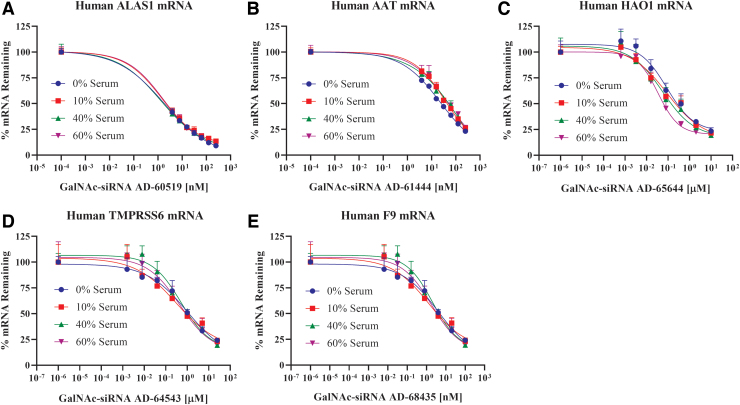
Minimal impact of human serum proteins on GalNAc-conjugated siRNA activity in human hepatocytes. Primary human hepatocytes were treated with increasing concentrations of AD-60519 **(A)**, AD-61444 **(B)**, AD-65644 **(C)**, AD-64543 **(D)**, or AD-68435 **(E)** by free uptake in the presence of varying concentrations of human serum (0%, 10%, 40%, or 60%, v/v). After 48 h, *ALAS1* **(A)**, *AAT* **(B)**, *HAO1* **(C)**, *TMPRSS6* **(D)**, or *F9* **(E)** mRNA levels, respectively, were assessed by RT-qPCR and percent of mRNA remaining was plotted. The range of the tested concentrations for each of these GalNAc-conjugated siRNAs was adjusted based on their varying potencies. Each data point is the mean of three replicates, which is represented relative to the average of the no GalNAc-conjugated siRNA treatment group (mock). Each percent serum treatment group was analyzed and compared with its respective mock. Error bars represent standard deviation. *AAT*, alpha-1 antitrypsin; *ALAS1*, aminolevulinic acid synthase; *F9*, coagulation factor IX; *HAO1*, hydroxy acid oxidase 1; mRNA, messenger RNA; *TMPRSS6*, transmembrane serine protease 6. Color images are available online.

Next, we investigated the potential impact of serum proteins on RNAi activity in primary human hepatocytes. At the time of seeding, GalNAc-conjugated siRNAs were added at varying concentrations, and for each siRNA concentration, the media were supplemented with 10%, 40%, or 60% human serum. Similar to the uptake experiments, after a 4-h incubation to allow free uptake of GalNAc-conjugated siRNAs through the *ASGPR*, the media was replaced with serum- and siRNA-free media, and on-target mRNA knockdown was assessed after 48 h. Five GalNAc-conjugated siRNAs against different targets were tested in this manner (Table 1). Treatment with each GalNAc-conjugated siRNA led to a concentration-dependent knockdown of the respective target mRNA in human hepatocytes when incubated with serum-free media, as expected (Fig. 2A–E). Importantly, increasing the percent human serum, up to 60%, minimally impacted the activity of each of these GalNAc-conjugated siRNAs. When incubated with 60% human serum, AD-65644 and AD-64543 at ∼3.2 μg/mL (200 nM) were ∼65% and 70% bound (or 35% and 30% unbound) to serum proteins, respectively (Table 4). At ∼6.5 μg/mL (400 nM), AD-65644 showed ∼50% ± 10% *HAO1* mRNA knockdown without human serum, while there was ∼70% ± 3% *HAO1* mRNA knockdown with 60% human serum (Fig. 2C). Similarly, AD-64543 at ∼3.2 μg/mL (200 nM) showed ∼28% ± 10% transmembrane serine protease 6 (*TMPRSS6*) mRNA knockdown without human serum and ∼30% ± 8% *TMPRSS6* mRNA knockdown with 60% human serum (Fig. 2D). Hence, in both cases, serum proteins did not negatively impact their RNAi activity. Thus, considered together, these data indicate that serum proteins have minimal impact on GalNAc-conjugated siRNA activity, which is consistent with the minimal impact observed on their uptake, *in vitro*.

## Discussion

Characterization of the extent of serum protein binding is an important aspect of conventional, small-molecule drug development because it can potentially have a significant impact on pharmacokinetic and pharmacodynamic properties. Protein binding properties of GalNAc-conjugated siRNAs and the potential impact on disposition and RNAi activity are expected to be different from that of small molecules because of their negatively charged hydrophilic nature and targeted *ASGPR*-mediated delivery to hepatocytes. Percent of GalNAc-conjugated siRNA bound to plasma proteins reaches up to 90% at clinically relevant plasma concentrations between 0.5 and 1 μg/mL (∼30–60 nM), and at concentrations greater than 1 μg/mL, percent binding decreases as serum protein binding saturates [11,12]. Consistent with these data, in this report, we showed that percent of GalNAc-conjugated siRNA bound to serum proteins reaches up to ∼80% when incubated with 100% FBS, at 4 μg/mL siRNA concentration. When GalNAc-conjugated siRNAs were incubated with 60% human serum, about ∼70% of GalNAc-conjugated siRNAs were bound to serum proteins. Under the tested conditions, we demonstrated that GalNAc-conjugated siRNAs maintain efficient *ASGPR*-mediated uptake and RNAi activity in hepatocytes.

In contrast to passive uptake of hydrophobic small molecules, the GalNAc ligand enables targeted delivery to hepatocytes through high-affinity interactions with the abundant and rapidly recycling *ASGPR* [6]. There are at least two potential reasons for why GalNAc-conjugated siRNA uptake would be unaffected by serum proteins. First, the binding affinity of the GalNAc ligand to *ASGPR* may be higher than that of bound serum proteins, resulting in minimal impact of serum on the overall efficiency of *ASGPR*-mediated uptake [13]. Second, it is possible that only the siRNA portion of the GalNAc-conjugated siRNA is interacting with serum proteins, allowing the GalNAc ligand portion to freely interact with the *ASGPR* and therefore have minimal impact on *ASGPR* binding and endocytosis. Indeed, while more extensive investigations are needed, in initial pull-down experiments with rat and human serum samples using biotinylated GalNAc, biotinylated GalNAc-siRNA, and biotinylated siRNA structural oligonucleotide information (Supplementary Table S1), we found that the majority of serum proteins bound to the siRNA portion and not to the GalNAc ligand (Supplementary Fig. S1). Even if siRNAs form large complexes with serum proteins, one of the primary functions of the *ASGPR* is to clear large macromolecules, including desialylated glycoproteins, prothrombotic components, hepatic lipoproteins, senescent platelets, and cellular fibronectin [15–17]. The molecular weights for some of these complexes can be up to ∼400 kDa, as is the case for cellular fibronectin [17]. Following uptake, it is conceivable that serum proteins bound to siRNA would be degraded in the endosomal compartment and thus the intracellular RNAi activity might be mediated by unbound siRNA. However, for a comprehensive understanding of these interactions, a proteomics study to identify serum proteins that bind to GalNAc-siRNA is warranted.

Additionally, when we assessed the activity of a GalNAc-conjugated 5-10-5 methoxyethyl gapmer antisense oligonucleotide (ASO) with a phosphorothioate backbone in human hepatocytes, we observed that its activity was also minimally affected in the presence of 60% human serum (Supplementary Fig. S2). It is well established that ASOs have a high propensity for protein binding due to their high phosphorothioate content [18–20]. This result further supports the hypotheses that the serum proteins that may be binding to the oligonucleotide portion of GalNAc conjugates do not impact *ASGPR*-mediated uptake into hepatocytes and that the GalNAc ligand is the primary determinant of uptake efficiency.

In conclusion, our data indicate that serum proteins have minimal impact on GalNAc-conjugated siRNA uptake and activity and thus potential differences in serum proteins across various human populations are expected to have minimal impact on pharmacokinetic and pharmacodynamic properties of this RNAi therapeutic platform.

## Supplementary Material

Supplemental data
