# Supplementary material for: Impact of Serum Proteins on the Uptake and RNAi Activity of GalNAc-Conjugated siRNAs
Source: Nucleic Acid Ther. 2021 Aug 4;31(4):309–15. doi: 10.1089/nat.2020.0919 (PMC8377513; doi:10.1089/nat.2020.0919)
Supplement: Supplemental data [file Supp_Data.docx]

**Supplemental Information** **Impact of Serum Proteins on the Uptake and RNAi Activity of GalNAc-conjugated siRNAs**

Saket Agarwal, Ruth Allard, Justin Darcy, Samantha Chigas, Yongli Gu, Tuyen Nguyen, Sarah Bond, Saeho Chong, Jing-Tao Wu, Maja M. Janas

Alnylam Pharmaceuticals, Cambridge, Massachusetts, USA

**Corresponding Author**

Maja M. Janas
Department: Early Development
Company: Alnylam Pharmaceuticals Inc.
Address: 675 W Kendall St, Cambridge MA, 02142
Phone: (617) 551-8255
Email: mjanas@alnylam.com

**Running Title:** Impact of serum on GalNAc-siRNAs

**Supplemental Materials and Methods**

**Test Materials and Oligonucleotide Synthesis**

The test articles, AD-73640 (biotinylated GalNAc-conjugated siRNA), AD-69542 (biotinylated unconjugated siRNA), A-140832 (GalNAc-conjugated ASO), and biotinylated GalNAc were synthesized by Alnylam Pharmaceuticals (Cambridge, MA). Structural oligo information can be found in Table S1.

**Serum Protein Pulldown**

Magnetic Streptavidin M-280 Dynabeads (Thermo Fisher Scientific) were washed twice with 1 mL of wash buffer (20 mM Tris pH 7.5, 120 mM NaCl, 5 mM CaCl_2_, 2.5 mM MgCl_2_, 0.5% Triton X-100), and incubated with 750 pmoles (50 µL of 15 µM solution in wash buffer) of biotin, biotinylated GalNAc, biotinylated siRNA (AD-69542), and biotinylated GalNAc-siRNA (AD-73640). Following a 30 minute rotation at room temperature, the beads were magnetically separated from the solution, and 50 µL of serum (BioIVT Lot RAT270490 or BioIVT Lot BRH 1029805) diluted 50% in wash buffer were added to the coated beads. Following a 30 minute rotation at room temperature, the beads were magnetically separated from the solution, washed twice with 1 mL of wash buffer, and the proteins were eluted by heating at 70^o^C for 10 minutes in 50 µL of 1.5X NuPAGE™ LDS Sample Buffer (Thermo Fisher Scientific) supplemented with 50 mM dithiothreitol. The elutions were analyzed by silver stain. Five microliters of each elution were loaded onto 20-well NuPAGE® 4-12% Midi Bis-Tris gel (Thermo Fisher Scientific) and electrophoresed in 1X NuPAGE® MOPS SDS Running Buffer at 120V. Silver stain was performed using Pierce™ Silver Stain Kit (Thermo Fisher Scientific) according to manufacturer’s instructions.

**Supplemental Tables**

**Table S1: Designs, sequences, and target mRNAs of the siRNAs and ASO used in the supplemental studies.**

| **Compound** | **Strand** | **Sequence (5 prime - 3 prime)** | **Target Accession Number** | |
| --- | --- | --- | --- | --- |
| **AD-73640** | S | QG•g•UuAaCaCGUuUuAgAuCaAL | N/A (non-targeting scrambled siRNA) | |
|  | AS | u•U•gAuCuAaAacgUgUuAaCc•a•g |  |  |
| **AD-69542** | S | QG•g•UuAaCaCGUuUuAgAuC•a•A | N/A (non-targeting scrambled siRNA) | |
|  | AS | u•U•gAuCuAaAacgUgUuAaCc•a•g |  |  |
| **A-140832** | AS | Ae•Ue•Ae•Ue•Ue•dT•dG•d^m^C•d^m^C•dT•dT•dT•d^m^C•dA•dT•Ue•Ge•^m^Ce•Ae•^m^CedAL | NM_000133 | |
| **L** |  | | |  |
| **Q** | 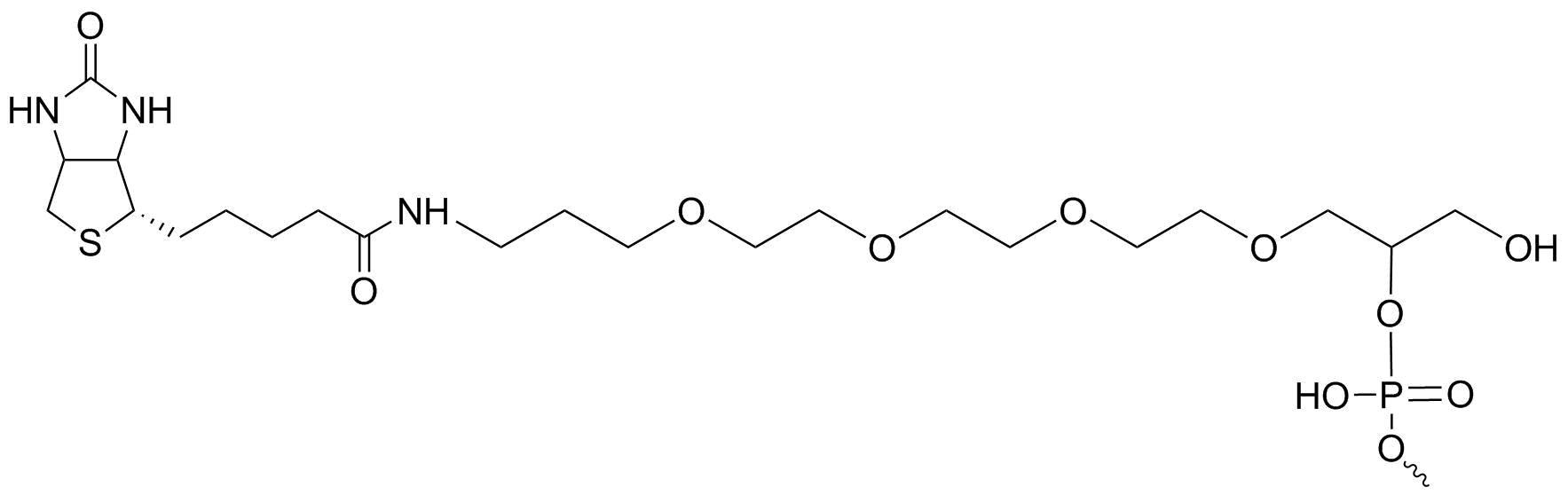 | | |  |

S and AS represent sense and antisense strands; upper-case and lower-case letters indicate 2'-deoxy-2'-fluoro (2′-F) and 2'-O-methyl (2′-OMe) ribosugar modifications, respectively; “d” before and “e” after upper-case letters indicates deoxyribosugar and 2’ methoxyethyl ribosugar modifications, respectively; ^m^C indicates methylated cytidine; • indicate phosphorothioate (PS) linkage; L indicates the trivalent *N*-acetylgalactosamine (GalNAc) ligand (structure above); Q indicates biotin ligand (structure above).

**Supplemental Figures**

**
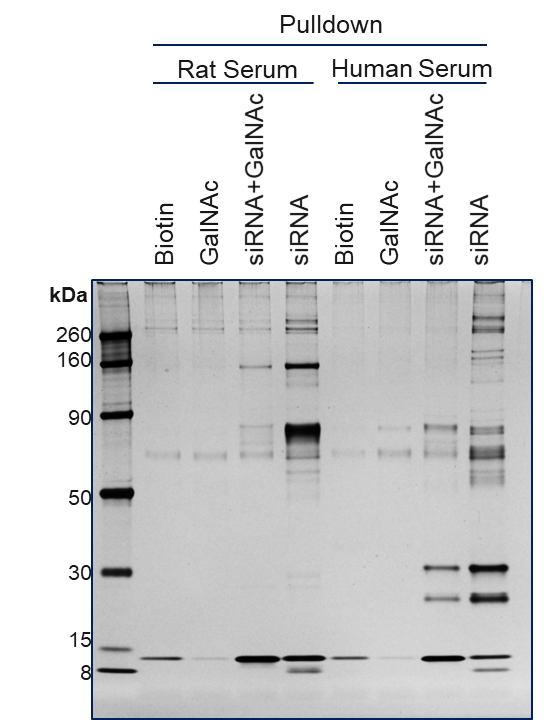
**

**Figure S1**: Minimal serum protein binding to GalNAc ligand alone, relative to unconjugated (AD-69542) or GalNAc-conjugated siRNA (AD-73640). Pulldown samples from rat and human serum using biotinylated GalNAc, biotinylated GalNAc-siRNA, and biotinylated siRNA analyzed using silver staining.

**Figure S2:** Minimal impact of human serum on GalNAc-conjugated ASO activity in human hepatocytes. Primary human hepatocytes were treated with increasing concentrations of A-140832 by free-uptake, in the presence of varying concentrations of human serum (0%, 10%, 40%, or 60%, v/v). After 48 hours, F9 mRNA levels were assessed by RT-qPCR and percent mRNA remaining was plotted. Each datapoint is the mean of three replicates which is represented relative to the average of no GalNAc-conjugated ASO treatment group (mock). Error bars represent standard deviation.
